# The Positive Impact of Donor Bone Marrow Cells Transplantation into Immunoprivileged Compartments on the Survival of Vascularized Skin Allografts

**DOI:** 10.1007/s00005-021-00631-8

**Published:** 2021-10-11

**Authors:** Arkadiusz Jundziłł, Aleksandra Klimczak, Erhan Sonmez, Grzegorz Brzezicki, Maria Siemionow

**Affiliations:** 1 Department of Regenerative Medicine, Cell and Tissue Bank, Ludwik Rydygier Medical College, Nicolaus Copernicus University in Torun, Bydgoszcz, Poland; 2 Department of Plastic, Reconstructive and Aesthetic Surgery, Collegium Medicum in Bydgoszcz, Nicolaus Copernicus University in Toruń, Bydgoszcz, Poland; 3 Hirszfeld Institute of Immunology and Experimental Therapy, Polish Academy of Sciences, Wrocław, Poland; 4 Department of Plastic Surgery, Cleveland Clinic, Cleveland, OH USA; 5 Katip Çelebi Üniversity, Atatürk Training Hospital, Plastic and Reconstructive Surgery Clinic, İzmir, Turkey; 6 Department of Neurosurgery, Virginia Commonwealth University, Richmond, VA USA; 7 Department of Orthopaedics, The University of Illinois at Chicago, Chicago, IL USA; 8 Department of Surgery, University of Medical Sciences, Poznan, Poland

**Keywords:** Bone marrow cells transplantation, Cells supportive therapy, Immunoprivileged compartments, Vascularized composite allotransplantation, Groin flap

## Abstract

Using the vascularized skin allograft (VSA) model, we compared the tolerogenic effects of different allogeneic bone marrow transplantation (BMT) delivery routes into immunoprivileged compartments under a 7-day protocol immunosuppressive therapy. Twenty-eight fully MHC mismatched VSA transplants were performed between ACI (RT1^a^) donors and Lewis (RT1^1^) recipients in four groups of seven animals each, under a 7-day protocol of alfa/beta TCRmAb/CsA (alpha/beta-TCR monoclonal antibodies/Cyclosporine A therapy). Donor bone marrow cells (BMC) (100 × 106 cells) were injected into three different immunoprivileged compartments: Group 1: Control, without cellular supportive therapy, Group 2: Intracapsular BMT, Group 3: Intragonadal BMT, Group 4: Intrathecal BMT. In Group 2, BMC were transplanted under the kidney capsule. In Group 3, BMC were transplanted into the right testis between tunica albuginea and seminiferous tubules, and in Group 4, cells were injected intrathecally. The assessment included: skin evaluation for signs and grade of rejection and immunohistochemistry for donor cells engraftment into host lymphoid compartments. Donor-specific chimerism for MHC class I (RT1^a^) antigens and the presence of CD4^+^/CD25^+^ T cells were assessed in the peripheral blood of recipients. The most extended allograft survival, 50–78 days, was observed in Group 4 after intrathecal BMT. The T cells CD4^+^/CD25^+^ in the peripheral blood were higher after intrathecal BMC injection than other experimental groups at each post-transplant time point. Transplantation of BMC into immunoprivileged compartments delayed rejection of fully mismatched VSA and induction of robust, donor-specific chimerism.

## Introduction

Vascularized composite allografts (VCA) represent a robust model for restoration for significant skin defects (Petit et al. 2003). However, skin and muscle components’ high antigenicity demands lifelong immunosuppression to prevent rejection and extend allograft survival (Siemionow 2020). The multiple side effects of lifelong immunosuppression supported the studies on the discovery of new tolerance-inducing strategies. Induction of donor-specific tolerance is a primary goal in VCA transplantation research to be applicable in the cases of non-life-threatening procedures (Cendales et al. 2008; Gordon et al. 2009). Various strategies to induce VCA tolerance have been discovered and tested (Siemionow and Nasir 2007). Allogeneic bone marrow cell (BMC) transplantation is a well-known option for the induction of donor-specific chimerism and prevention of allograft rejection in the experimental models (Asari et al. 2011; Leonard et al. 2013b; Safinia et al. 2013; Siemionow and Nasir 2008). However, the effects of cell-based therapies the following transplantation into immunoprivileged compartments has not been yet well-established as a promising new approach for tolerance induction strategy. The immunoprivileged sites are more suitable for cellular transplants and offer complete or partial protection from all rejection without the need for life-long immunosuppressive therapy (Stevenson et al. 1997). Previously, intrathecal, intracapsular space, and intermembrane testis spaces have been suggested as immunoprivileged regions able protecting allogeneic cells from rejection (Fijak et al. 2011; Muldoon et al. 2013; Robertson et al. 2007). However, limited investigations have been conducted evaluating the implications of regional cell transplantation on the induction of donor chimerism. Moreover, there is limited number of studies in the literature on the impact of BMC transplantation into the immunoprivileged regions and its relation with the allograft survival or rejection. Thus, we introduced our well-established VCA transplant model to investigate a new tolerogenic approach of different routes of bone marrow transplantation (BMT) into immunoprivileged compartments of vascular skin allograft recipients.

## Materials and Methods

### Animals and Animal Care

Cleveland Clinic’s Institutional Animal Care and Use Committee (Cleveland, OH, USA), accredited by the American Association for the Accreditation of Laboratory Animal Care (#2012-0841), approved this study. All animals received humane care in compliance with the “Principles of Laboratory Animal Care” formulated by the National Society for Medical Research and the “Guide for the Care, and Use of Laboratory Animal Resources” published by the US National Institutes of Health (Guide for the care and use of laboratory animal resources 2011). Animals were caged at room temperature on a 12-h light/dark cycle. Standard laboratory food and water were available ad libitum. Animals were housed in a barrier animal facility and cared for according to specific National Institutes of Health animal care guidelines. Inbred 8–10 week-old Lewis (LEW, RT1^1^) rats weighing between 200 and 225 g and 4–6 week-old ACI (RT1^a^) rats weighing between 100 and 125 g were purchased from Harlan Sprague Dawley (Indianapolis, IN) fully MHC mismatched. In all allotransplantations, LEW rats were recipients, and ACI rats served as allograft donors. Surgical procedures were performed under an anesthesia cocktail of ketamine (30 mg/kg), xylazine (6 mg/kg), and acepromazine (1 mg/kg). Additional doses were given if necessary. Postoperative pain was controlled by applying non-steroidal anti-inflammatory drugs, paracetamol, and opioids (McPherson 1980).

### Experimental Groups

Twenty-eight fully MHC mismatched vascularized skin allograft (VSA) transplants were performed between ACI (RT1^a^) donors and Lewis (RT1^1^) recipients in four groups of seven animals each, under a 7-day protocol of alfa/beta TCR/CsA therapy (Siemionow et al. 2002, 2003). VSA transplants were supported by donor ACI (RT1^a^) BMT in the amount of 100 × 10^6^ cells transplanted into three different immunoprivileged compartments; Group 1 served as allograft rejection control without BMC. VCA therapy groups received bone marrow cells (100 × 10^6^) transplantation into three different immunoprivileged compartments: the intracapsular (Group 2), the intragonadal (Group 3), and the intrathecal space (Group 4).

### Surgical Procedure

There was a two-stage operating procedure:

#### Vascular skin allograft transplantation procedure

**Fig. 1:**
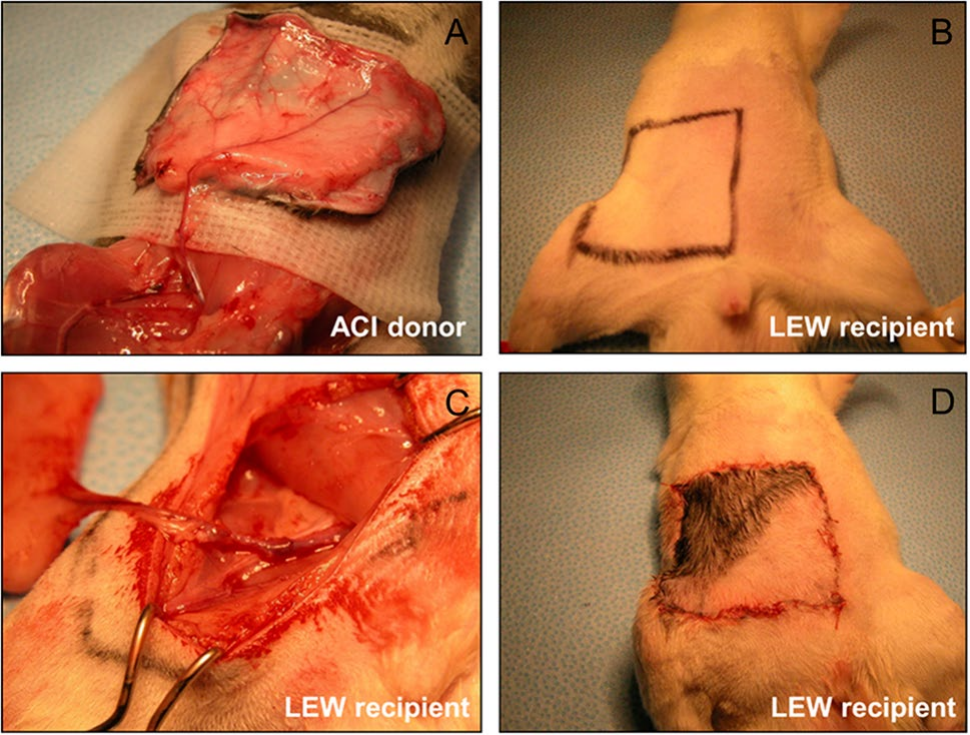
Transplantation procedure of VSA groin flap transplantation. Explanation in a text

The surgical procedure of skin allograft transplantation was accomplished in two stages: harvesting the flap and recipient preparation and allograft transplantation (Fig. 1). The donor VSA was approximately four by 4 cm. The border of the VSA was extended from 5 cm below the xiphoid process and the last rib (half the length of the symphysis pubis) to the inguinal ligament and between the right mid-axillary line and midline. Following the skin island’s incision, the right VSA was elevated over the anterior abdominal wall muscle. Superficial epigastric vessels were dissected to their origins at the femoral artery (Siemionow and Kulahci 2007). Femoral vessels were ligated proximally near the inguinal ligament and distally from the region of epigastric vessel origins and divided during flap elevation (Nasir et al. 2008) (Fig. 1A). In the recipient rat Lewis (RT1^1^), the suitable femoral vessels were exposed and dissected proximally up to the inguinal ligament and distally down to the branching from the superficial epigastric vessels. In the recipient rat the groin skin was resected to make the defect proportional to the donor flap size (Fig. 1B). The flap ACI (RT1^a^) pedicle was anastomosed with the femoral artery. The recipient’s vein using standard end-to-end 10–0 nylon interrupted sutures under operating-microscope magnification with conventional microsurgical techniques (Fig. 1C). The graft’s skin component was sutured to the edges of the previously created skin defect in the recipient groin region with absorbable sutures 4–0 (Fig. 1D) (Vicryl, Ethicon, Inc.).

#### Bone marrow cells (BMC) transplantation into immunoprivileged compartments (Fig. 2)

**Fig. 2:**
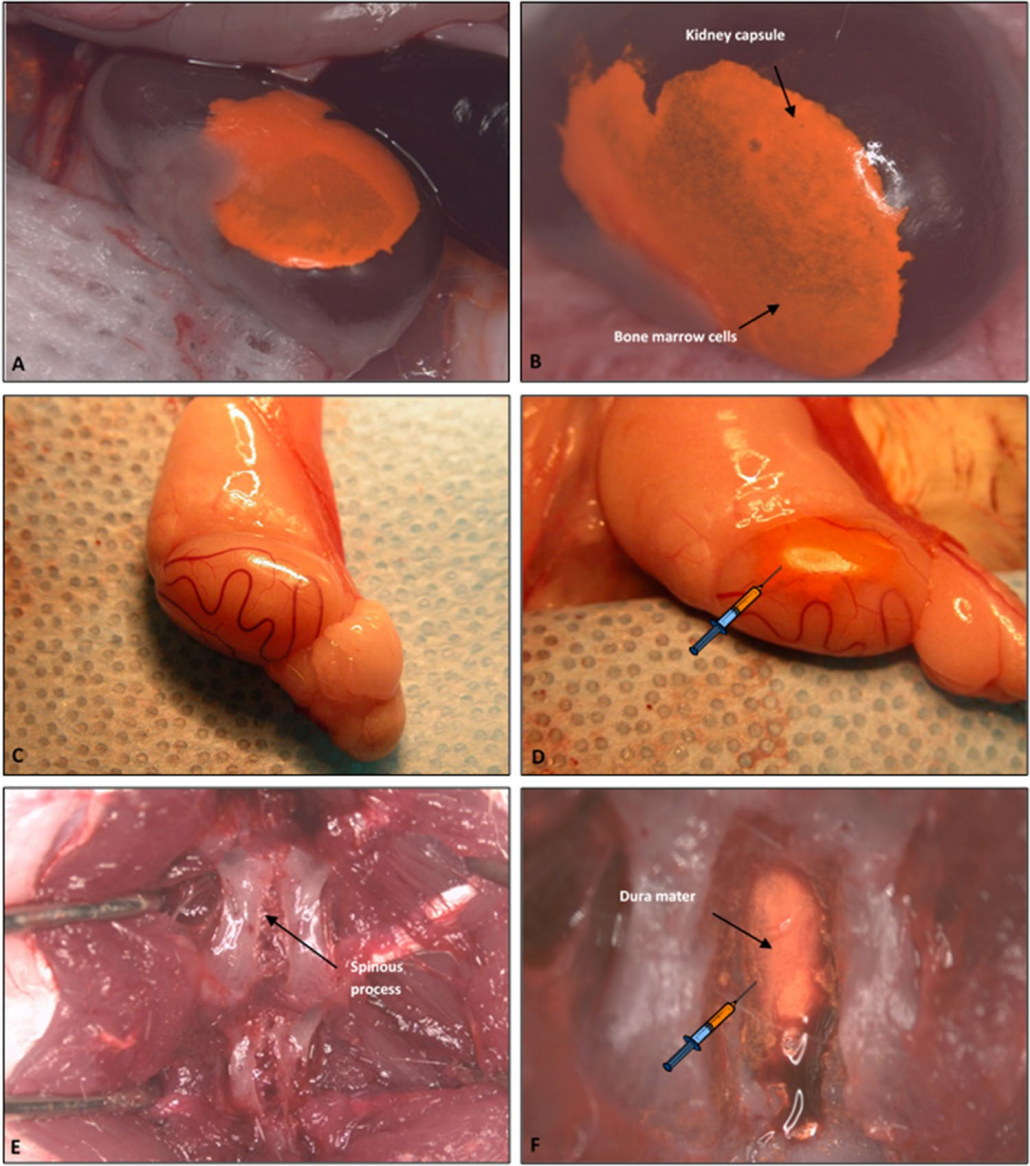
Different routes of bone marrow cells (BMC) transplantation (100 × 10^6^). **A**, **B** Group 2: Intracapsular BMC transplantation; **C**, **D** Group 3: Intragonadal BMC transplantation; **E**, **F** Group 4: Intrathecal BMC transplantation

##### *Group 2: Intracapsular BMC transplantation* (Fig. 2A, B)

After anesthesia has taken effect, two saline-dampened cotton-tipped applicators are used to maneuver through a small incision through the skin, muscle, and peritoneum of the animal’s left backside expose the left kidney outside of the body. Employing a slight pressure to both sides of the incision, we rolled the kidney out of the abdominal cavity. The kidney capsule was moistened by applying saline with a cotton-tipped swab. Then through the lower pole of the kidney, not involving the renal pelvis, 0.06 ml of bone marrow cell suspension was injected under the upper kidney capsule using a 0.5 ml syringe with a 31G needle (Robertson et al. 2007; Toledo-Pereyraet al. 1984). To stop the bleeding, a dry cotton-tipped swab or cauterization with low heat was used. Then, the kidney was pre-moistened with sterile saline. The kidney was gently placed back into the peritoneum before closing the abdominal wall. The muscle layer and the skin were sewed with running 4–0 absorbable sutures.

##### *Group 3: Intragonadal BMC transplantation* (Fig. 2C, D)

The animal was placed in the lateral supine position. Then the scrotal and inguinal regions were trimmed and swabbed with a Povidone–Iodine solution before the surgery. A linear incision was made lateral to the median raphe on the right side. The right testis enclosed in the parietal vaginal tunic was gently exposed using a cotton-tipped stick. The small incision helped to keep the testicle raised and exposed. The testicle was swabbed with normal saline preventing tunica albuginea rupture. Using a 0.5 ml syringe with a 31G needle, the 0.06 ml of bone marrow cell suspension was injected between the tunica albuginea and convoluted seminiferous tubules (Fijak and Meinhardt 2006; Schlatt et al. 1999). After the cell transplantation, the testicle was placed back in the scrotum. The scrotum skin was closed with a running stitch using 5–0 running silk sutures and a C-6 19 mm needle.

##### *Group 4: Intrathecal BMC Transplantation* (Fig. 2E, F)

The rat was placed in a prone position, and a midline skin incision was performed approximately 2–3 cm above vertebrae L4–L5. The paraspinous muscles were detached using a thermocautery technique. Using the Love–Kerrison punch, the laminotomy was conducted. Particular attention was paid to avoid damaging the external vertebral venous plexuses (extraspinal veins) responsible for persistent and massive venous hemorrhage. Afterward, dura mater was exposed, and 0.08 ml of bone marrow cell suspension was transplanted using a 0.5 ml syringe with a 31G needle (Glinkowski and Ciszek 2000; Muldoon et al. 2013). This stage requires meticulous attention as rapidly injected fluid in the subarachnoid space could induce cerebrospinal fluid hypertension, potentially leading to the irreversible complications. The needle was gently removed, and the needle hole was covered in dura mater with the previously prepared paraspinous muscle patch and silked with bone wax if necessary. Subcutaneous tissue and skin were adapted by running 4–0 absorbable sutures.

### Preparation of Donor BMC

According to our well-established technique, bone marrow cells were prepared from the ACI(RT1a) rat femoral bones (Klimczak et al. 2007). Before transplantation, BMC was labeled with red fluorescent membrane dye PKH-26 (Sigma–Aldrich, UK) in the Diluent C buffer solution for 5 min. Labeling was stopped by incubation with 1% bovine serum albumin in phosphate-buffered saline (PBS) for 1 min and complete α-MEM medium. PKH-26 labeled BMC were prepared to a final concentration of 100 × 10^6^ cells (Tario et al. 2007).

### Immunosuppressive Protocol

In all treatment groups, alpha/beta-TCR monoclonal antibodies (mAb) and Cyclosporine A (CsA) (Novartis, East Hanover, NJ) were administered 1 h before surgery. CsA was injected subcutaneously (16 mg/kg/day), and intraperitoneal injection of alpha/beta-TCR mAb (250 mg/day) (clone R73, Pharmingen, San Diego, CA) was administered simultaneously with CsA during the 7-day protocol. The efficacy of immunosuppressive drug administration was monitored by the serum level of CsA and alpha/beta-TCR checking simultaneously with blood sample harvesting (Siemionow et al. 2005a).

### Clinical Evaluation of Graft-versus-Host Disease

The animals’ general health and weight were monitored throughout the study. According to the previously published criteria, recipients were clinically evaluated for the presence of the graft-versus-host disease (GVHD) signs, including unkempt appearance, diffuse erythema (particularly mucosa and ear), hair loss, diarrhea, and rash of paws or snout, and failure to thrive (Kanitakis 2008). Diagnostics included daily measurement of body weight during the first month and then at weekly intervals.

### Assessment of Transplant Viability and Histological Evaluation

**Fig. 3:**
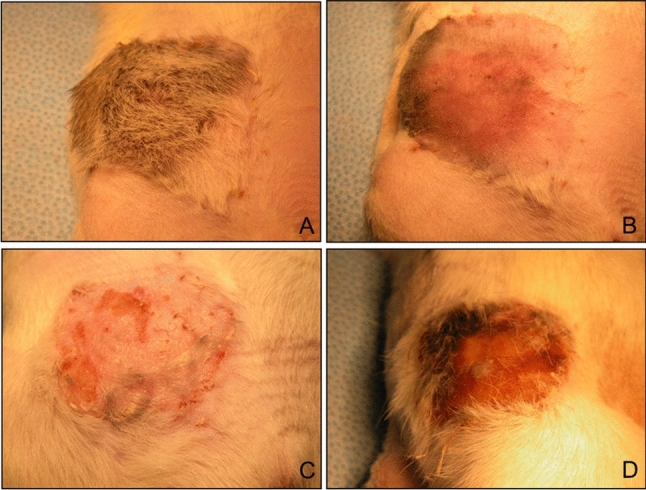
Clinical assessment of VSA rejection in Group 4 after intrathecal BMT: **A** no signs of rejection at day 35; **B** inflammation phase at day 58; **C** progression of rejection at 63 days, **D** necrosis: complete rejection at day 72 post-transplant

The skin island’s viability was assessed by observing the skin allograft transplants by the same surgeon (AJ). The survival of the VSA skin transplant was evaluated during the postoperative follow-up period by assessing the flap color, edema, erythema, hair loss, and necrosis compared with healthy skin (Fig. 3). Skin “punch” biopsies from the donor VSA and the contralateral skin recipients 21, 35, and 63 days after transplantation were performed unless rejection onset was identified. After clinical symptoms indicating skin rejection emerged, we collected additional skin biopsies from both the VSA and healthy skin from the recipient groin area for better collation. To evaluate the histological grade of acute rejection, the Buttemeyer standard scale was used: Stage 0—the correct appearance of the epidermis with no signs of rejection; Stage 1—focal infiltration of mononuclear cells, vacuolation of the basal layer of the epidermis; Stage 2—blistering suprabasal layer and mixed cellular infiltration; Stage 3—marked edema, vasculitis, and necrosis (Büttemeyer et al. 1996). At the end of the observation, we harvested the necrotic transplanted flap of skin from the donor ACI and an unoperated portion of the Lewis’ recipient skin.

Additionally, the liver, spleen, kidney, thymus, and three to four lymph nodes from the front of the neck were collected for further evaluation. For histological examination, graft and recipient tissues were fixed in 10% neutral buffered formalin (Sigma–Aldrich, USA) and embedded in paraffin before tissue sectioning and H&E staining. A pathologist evaluated the histology slides under a light microscope for the viability of skin, muscle, and bone, for the presence of hematopoietic cells, and for signs of fibrosis and rejection. Two pathologists blinded to the group assignment performed the assessments.

### Evaluation of BMC for the Presence of Donor-Specific Chimerism

Peripheral blood samples were taken at day 7, 21, 35, and 63 days post-transplantation from the external jugular vein. Additional blood samples were taken at clinical signs of rejection. For assessment of ACI donor chimerism, combinations of conjugated mouse anti-rat RT1^a^-FITC (for MHC class I of donor cells, clone C3, BD Pharmingen) with CD4-PE (clone OX-35), CD8a-PE (clone OX-8), and CD45RA-PE (clone OX-33) were used. After incubation, a lysing solution was used, and then samples were fixed with a 1% paraformaldehyde (PFA) solution. Opposing control panels were tested and included isotype-matched antibodies IgG_1_-FITC/IgG_2_-PE and PBS samples. The analysis was performed on 1 × 10^4^ cells, using FACS SCAN (BD Pharmingen) and FlowJo software (Siemionow et al. 2005b, 2008).

#### *Assessment of Engraftment of PKH-26* + *Stained Donor-Origin Cells by Immunofluorescence Analysis*

Cell trafficking potential and engraftment of donor-origin cells into lymphoid and non-lymphoid organs of VSA allograft recipients was assessed based on immunofluorescence signal delivered from PKH-26 labeled cells. Frozen tissue samples of lymph nodes, thymus, and skin punch biopsies of both donor and recipient were harvested randomly during blood sample harvesting on 7, 21, 35, and 63 days post-transplantation. Also, partial splenectomies were performed at 35 and 63 days after transplantation to one representative in each Group. Tissue sections were cut into 4 μm sections and dried for 40 min at room temperature, then fixed in cold (– 20 °C) acetone for 10 min and rinsed three times for 5 min in PBS. DAPI solution (Vectashield mounting media) was applied to each sample. The specimens were analyzed for the presence of PKH-26 positive cells using a fluorescence microscope.

### Statistical Analysis

Results were reported as mean ± standard deviation (SD). The VSA survival rates in treatment groups were evaluated by the ANOVA method. The level of chimerism (ACI(RT1^a^), CD4/(RT1^a^), CD8/(RT1^a^), CD45RA/(RT1^a^), presence of CD4^+^/CD25^+^ T cells, and efficacy of the immunosuppressive treatment was compared by Statistica 9.0 and Student’s *t* test. The significance of changes in the studied chimerism parameters in time was assessed by *t* test. Differences between groups were considered significant at *p* < 0.05 (Domański 1979).

## Results

### Assessment of GVHD

There was no decrease in body weight compared to the preoperative level. All animals were in good general condition. There were no characteristic GVHD symptoms, such as hair loss and skin color, specifically with changes around the ears, diarrhea, or shortness of breath. None of the animals showed any sign of GVHD during follow-up examination. Moreover, there was no confirmation of GVHD in histology assessment. All wounds healed initially, and there was no postoperative wound infection. There were no complications associated with BMC transplantation procedures into immune-privileged regions.

### Clinical Assessment of VSA Transplants

All animals survived the early postoperative period without any complications, and all wounds healed. There were no postoperative wound infections or complications associated with bone marrow donors’ transplantation into recipient immunologically privileged compartments. The patency of vessels supplying the transplanted vascularized flap was confirmed by intraoperative macroscopic examination during blood sampling at 21 days post-transplantation procedure.

**Fig. 4:**
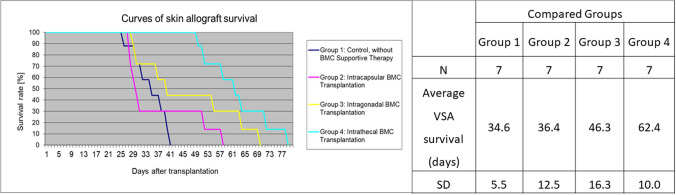
Skin allograft survival time. Group 1: Control, without BMC supportive therapy; Group 2: Intracapsular BMC (100 × 10^6^ cells) transplantation; Group 3: Intragonadal BMC (100 × 10^6^ cells) transplantation; Group 4: Intrathecal BMC (100 × 10^6^ cells) transplantation. The controls without cellular therapy rejected VSA between 26 and 41 days after transplantation. Allografts in Group 2 and Group 3 were rejected between 28–57 and 29–69 days, respectively. The longest allograft survival of 50–78 days was achieved in Group 4 after intrathecal BMT transplant

The controls without cellular therapy rejected VSA between 26 and 41 days (34.6 ± 5.5) after the transplantation procedure. Allografts in Group 2 and Group 3 were rejected between 28–57 (36.4 ± 12.5) and 29–69 (46.3 ± 16.3) days, respectively (Table 1). The most prolonged allograft survival, 50–78 (62.4 ± 10) days, was achieved in Group 4 after intrathecal BMT (Fig. 4). Average allogeneic skin flap survival was statistically significant in Groups 4 and 3 (*p* = 0.001 and *p* = 0.002) compared with control Group 1. In contrast, median survival in Group 2 was not statistically different from Group 1. The longest-individual VSA survival was observed in Groups 4, 3, 2, and 1, respectively, consistent with average survival time in different groups. VSA skin flaps’ survival time in Group 4 was significantly longer than Group 1 and 2 (*p* = 0.001 and 0.002, respectively). The survival curves for all experimental groups are plotted in (Fig. 4).

**Table 1:** The onset of VSA rejection

	Comparison of rejection between groups			
	Group 1	Group 2	Group 3	Group 4
*N*	7	7	7	7
Average onset of VSA rejection (days)	28.43	28.00	38.57	49.14
Average duration of the rejection process (days)	6	8	8	12
SD	7.59	12.15	15.31	9.30

Group 1: Control, without BMC supportive therapy; Group 2: Intracapsular BMC (100 × 10^6^ cells) transplantation; Group 3: Intragonadal BMC (100 × 10^6^ cells) transplantation; Group 4: Intrathecal BMC (100 × 10^6^ cells) transplantation. Noteworthy is the different length of the rejection process in individual groups. Longest average rejection was observed in Group 4 (12 days) and the shortest time of rejection was observed in Control Group 1 (6 days)

### Histological Examination of VSA

**Fig. 5:**
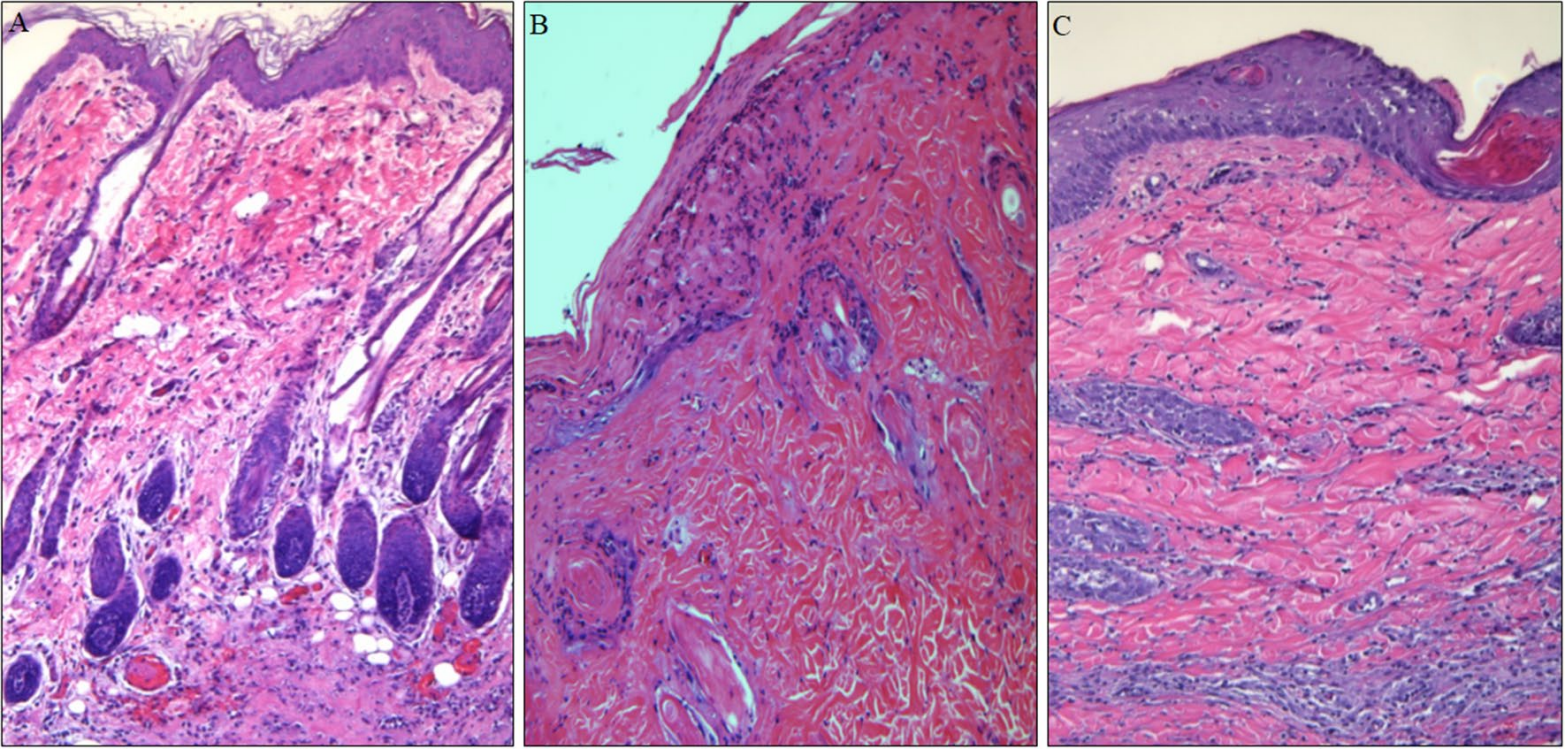
Hematoxylin and eosin staining of the skin samples during the rejection process, presenting the dynamics of the allogeneic skin flap rejection process in a Control Group I. The samples represent a rejection of VSA flaps after 14 days—Grade I (**A**), 21 days—Grade II (**B**), and Grade III—32 days after transplantation. Magnification (× 400)

In all groups, skin biopsies were taken from the donor VSA the first week after transplantation, and recipient skin biopsies revealed no histological changes and rated grade 0 on a rejection scale (Fig. 5).

In allograft rejection controls (no BMT), flaps showed clinical signs of rejection after 14 days post-transplantation. It was 7 days after immunotherapy cessation with single focal infiltrates of mononuclear cells with moderate infiltration of lymphocytic cells in the dermis upper layer-grade 1 Buttemeyer’s scale. However, in other groups simultaneously, skin biopsies showed normal epidermis and dermis (grade 0 on the rejection scale). All flaps showed clinical signs of completed rejection between 26 and 41 days post-transplant in allograft rejection controls. All skin graft biopsies were randomly taken from the animal of each Group, respectively. Group 2 (intracapsular Group) revealed histopathological third-degree rejection symptoms with clearly marked necrosis of the epidermis with mixed cell infiltration involving the entire thickness of the skin and subcutaneous tissue with small diameter perivascular inflammatory process between 28 and 57 days post-transplantation procedure. Skin biopsies taken from VSA in Groups 3 and 4 during the first clinical symptoms of rejection revealed epidermis focal infiltrates composed of mononuclear cells with little infiltration in the initial dermis stage rejection (average onset 38.57 vs. 49.14). Monitoring the rejection process assessed on the skin allograft biopsies taken from each experimental Group allowed us to conclude that the dynamics of the VSA rejection process were much slower in the intrathecal Group when compared with the other Groups. The most protracted average lasting VSA rejection process was observed in Group 4 (12 days), then in Group 3 (8 days) and Group 2 (8 days) (Table 1). The rejection process's duration was the most aggressive in Group 1, lasting six or fewer days.

### Engraftment of the Donor Origin Cells into the Lymphoid and Non-lymphoid Tissues of the Recipient

**Fig. 6:**
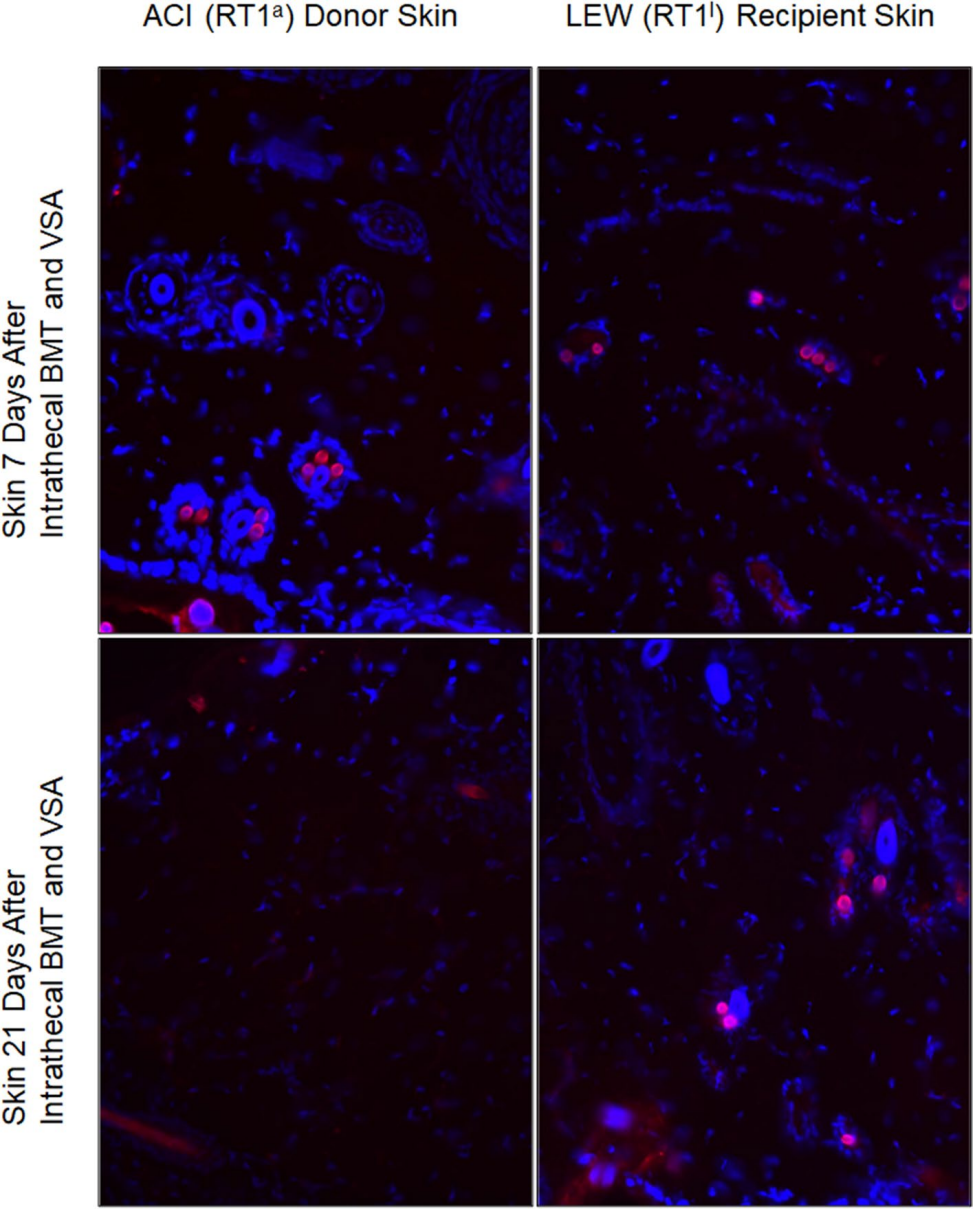
Immunohistological assessment of the engraftment of PKH stained BMC in different time points after intrathecal bone marrow injection with simultaneous VSA transplantation

Engraftment of donor-origin cells, identified by PKH-26 expression, at evaluation time points (7, 21, 35, and 63) post-transplant was confirmed in the spleen and lymph nodes, but not in the thymus of VSA allograft recipients. In the spleen, numerous donor-derived cells were localized. However, no significant differences in donor cell redistribution intensity were observed in tissue among evaluated groups. Both donor and recipient skin biopsies revealed PKH-26+ stained cells. Moreover, specific accumulation of donor cells, an average of two to three per one hair follicle in the dermis, was observed. The highest cell engraftment in airfoil vascularized and contralateral native skin recipients were evaluated in Group 4 after intrathecal BMC transplantation at 7 and 21 days post-transplantation (Fig. 6).

### Flow Cytometry for Determination of Donor-Specific Chimerism

**Fig. 7:**
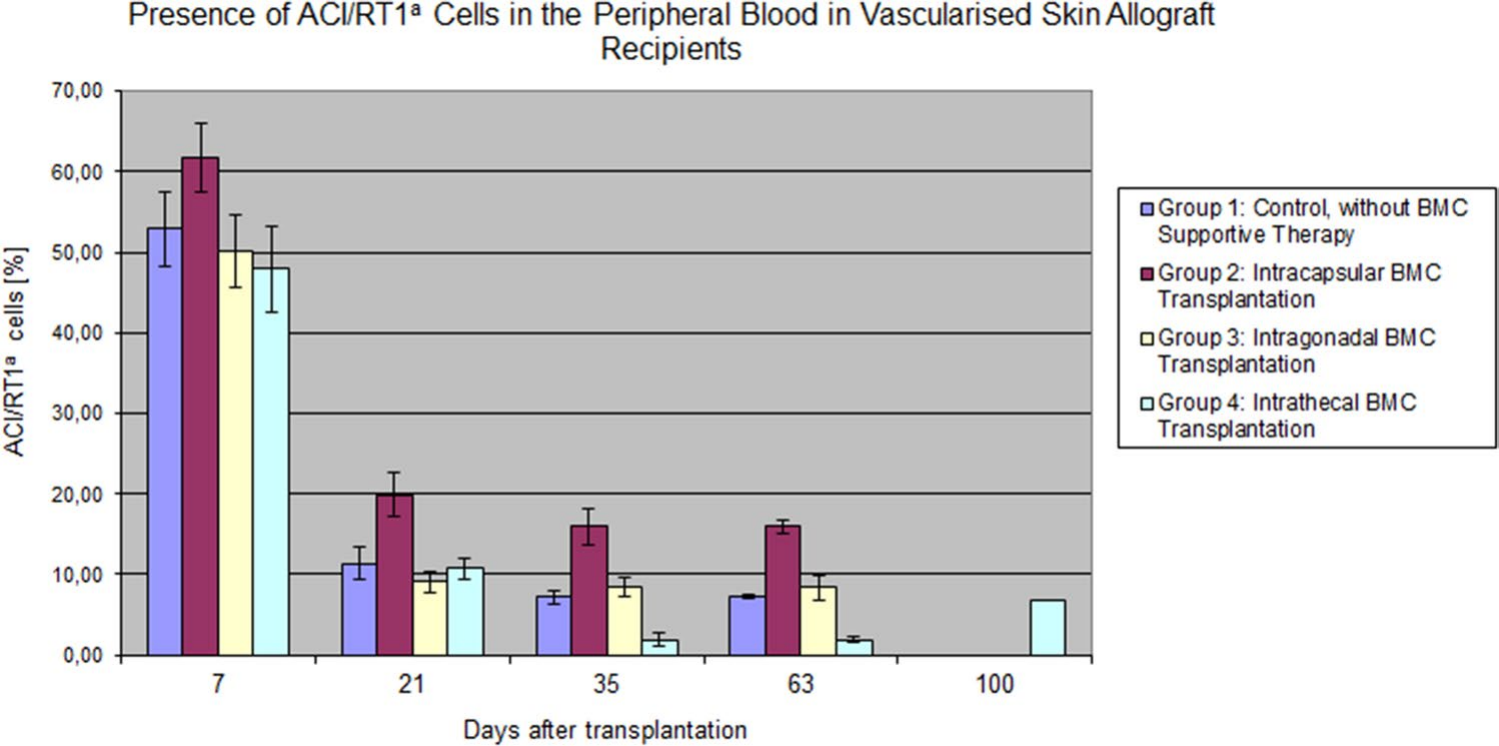
Comparison of the ACI/RT1a donor-specific chimerism in the peripheral blood of the recipient. Total chimerism was calculated as the sum of the T cells (RT1a/CD4, RT1a/CD8), B cells (RT1a/CD45RA), and monocyte/granulocyte (RT1a/CD11b/c) lymphocytes and is presented as mean values with the standard deviations. The peak in chimerism values was observed on day 7 after donor bone marrow transplantation. The highest values were reveled in the Group after administration of BMC cells under the renal capsule at each observation time. In all groups, chimerism decreased over time until day 63 post-transplant. The lowest values were observed between days 35 and 63 post-transplant in the Group receiving intrathecal bone marrow administration to the cerebrospinal fluid. In the intrathecal Group, there was an increase in the total value of the RT1a chimerism up to 6.85% on day 78 post-transplant

The multilineage donor-specific chimerism (ACI, RT1^a^) was assessed as the sum of donor T lymphocytes (RT1^a^/CD4 and RT1^a^/CD8) and B (RT1^a^/CD45RA) cells. The chimerism values are presented in the figure (Fig. 7). The multilineage donor-specific chimerism (ACI, RT1^a^) in all active groups followed a hyperbolic descending curve course as a function of time. The highest level of donor specific chimerism was observed seven days following BMT in each treatment group compared to the other time points, respectively. During the follow-up period, the peripheral blood’s highest chimerism level was found in the intracapsular Group (Group 2). The assessment of total donor chimerism RT1^a^, presented in Fig. 7, indicates that 2 months after the transplantation procedure, the highest level of chimerism was detected in the intracapsular Group 2 (7.21%).

In contrast, the lowest level of chimerism was found in the intrathecal Group 4 (1.92%). In all groups, the chimerism level gradually decreased over the entire follow-up period; however, slight increases was noticed in the intracapsular and intrathecal Groups on day 63 and day 78, respectively. The lowest chimerism levels were observed between days 35 and 63 in Group 4, where bone marrow cells were transplanted into the cerebrospinal fluid via intrathecal injection. However, in Group 4, after 78 days, the total value of the donor chimerism (RT1^a^) increased to 6.85%. Assessment of chimerism kinetics showed a sharp decline of 40% in each cell subpopulation between 7 and 21 days after transplantation.

**Fig. 8:**
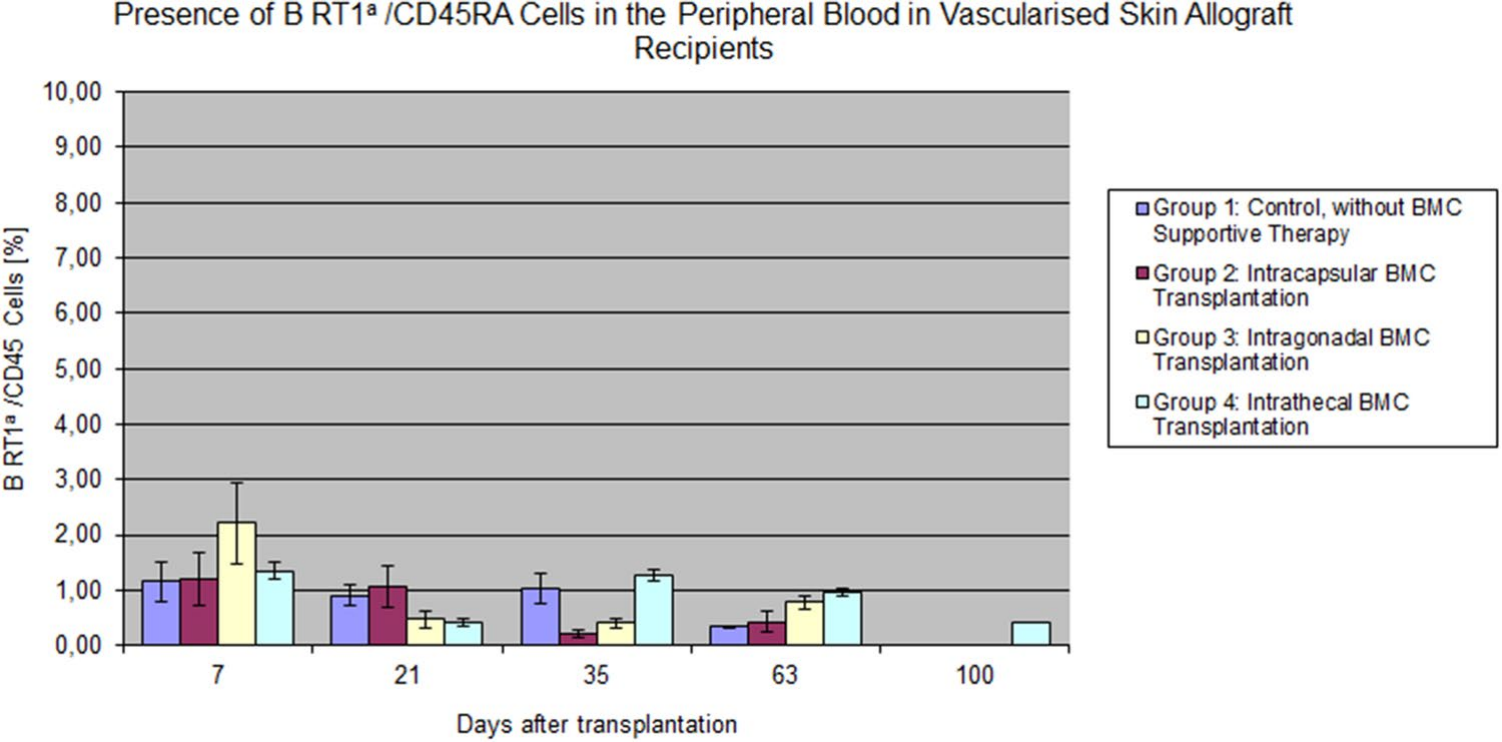
The presence of RT1a/CD45RA B lymphocytes in the peripheral blood of the VSA recipients. In Group 3, the mean values for the B cells (RT1a/CD45RA) at 7 days after transplantation exceeded 2%. Despite the decrease in B lymphocytes levels in Group 4 at day 21 post-transplant, we have observed chimerism stabilization at the level around 1%

The mean value of B cells (RT1a/CD45RA) at 7 days after transplantation in Group 3 exceeded 2% and over time B cell values ranged between 0 and 1.05%, except for day 35 in Group 4, where B cell chimerism value exceeded 1.26% and was significantly higher than on day 21–0.42%. Besides day 21, the level of B cells (RT1a/CD45) of donor-origin was stable only in Group 4. Moreover, there were differences at day 21 between groups 1 and 3 and 1 and 4 *p* < 0.047 and *p* < 0.007, respectively. On day 35, statistically significant differences were noted between Groups 1 and 3 and 2 and 4, *p* < 0.008 *p* < 002, respectively. In all groups, the mean values were lower than in the control group (Fig. 8).

**Fig. 9:**
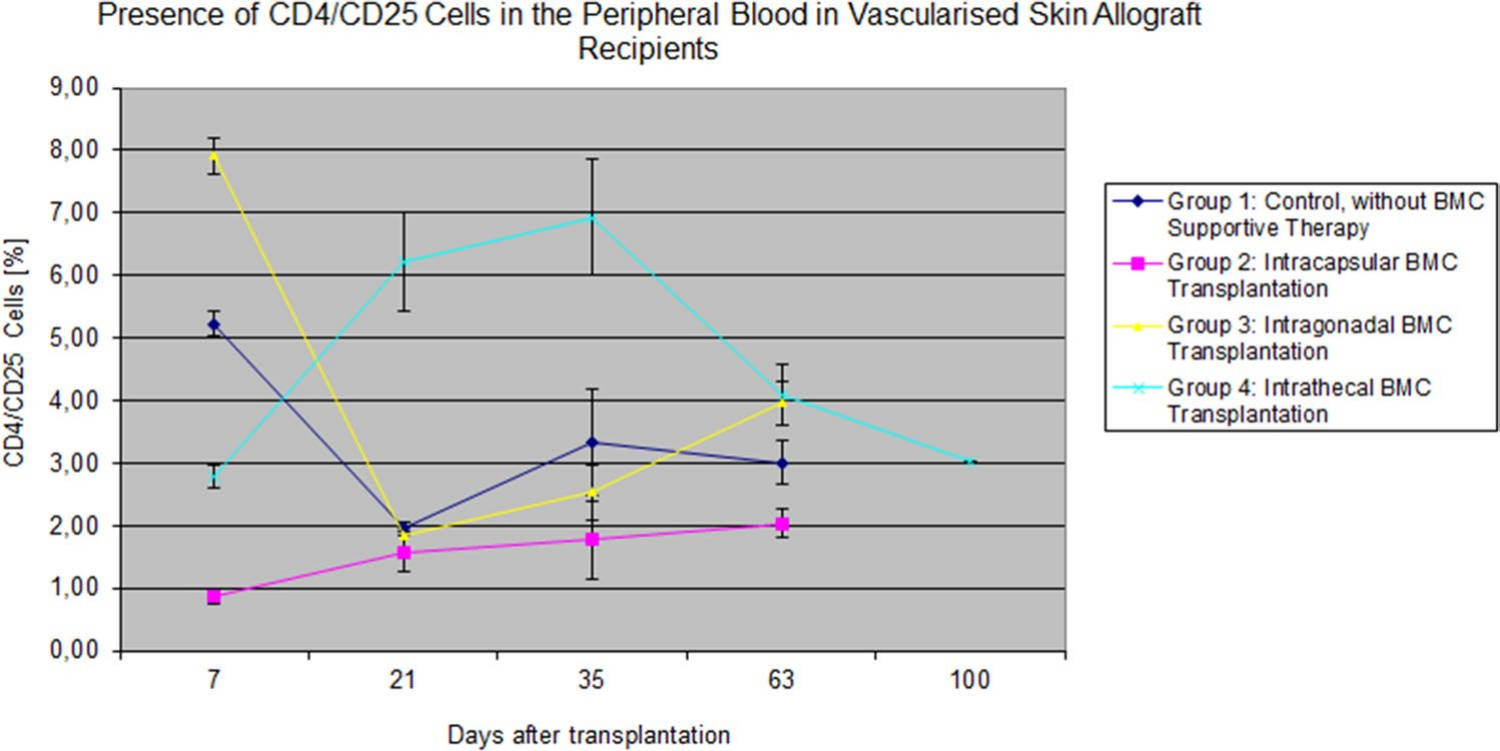
The presence of the Treg cells in the peripheral blood of the recipient. The level of Treg cells RT1^a^ CD4/CD25 in the peripheral blood was higher after the intrathecal injection compared to the other groups at each time point after transplantation (*p* < 0.05)

The highest T cell CD4^+^/CD25^+^ (7.91%) level was found on day 7 in the intragonadal injection group. However, the highest levels of CD4^+^/CD25^+^ T lymphocytes at days 21 and 35 were observed at the intrathecal Group, 6.23% and 6.93%, respectively (Fig. 9.). The significant differences in T lymphocytes CD4^+^/CD25^+^ values were noticed on 21 days between the control and intrathecal Group 2.12% vs. 6.24% (*p* < 0.0002). In Groups 1, 2, and 3, the number of CD4^+^/CD25^+^ cells revealed a decrease over time. In group 4, there was an increasing trend in CD4^+^/CD25^+^ cells up to 35 days post-transplant, followed by a decrease starting at day 63 post-transplant (Fig. 9). Comparison of the CD4^+^/CD25^+^ T cells values as the function of time revealed significant decreased of CD4^+^/CD25^+^ T cell in Group 1 and 3 (*p* < 0.02 and *p* < 0.03, respectively), with no statistically significant differences observed over time in Group 2 and 4 (*p* = 0.25, *p* = 0.16).

## Discussion

The main goal of transplant surgery is to achieve organ acceptance without lifelong immunosuppressive therapy to prevent transplant rejection (Siemionow 2017). The immunosuppression side effects are the most limiting factor for routine clinical application of VCA and present the critical argument from an ethical perspective against use of allograft transplantation in the procedures that improve quality of life rather than are saving the life (Morelon et al. 2012). In the last 2 decades a significant progress was made in the application of the non-myeloablative protocols and by introduction of a new immunosuppressive drugs to induce tolerance to in VSA (Hivelin et al. 2016). Numerous studies confirmed the beneficial role of donor-specific chimerism in allograft survival, but other reports suggest no connection between chimerism and tolerance. Therefore, investigations on non-animal models are conducted to acknowledge this correlation (Hartung and Corsini 2013; Schnider et al. 2013). It was recognized that the skin manifests the highest immunogenicity of all CTA components, confirmed in the classification of skin-containing composite tissue allograft (Cendales et al. 2006; Leonard et al. 2013b). This survey is complicated because the CTA’s transplantation (Schnider et al. 2013). This essential fact highlights the necessity of pursuing new alternative tolerance induction protocols (Leonard et al. 2013a). The one promising method for reducing or eliminating lifelong immunosuppression is chimerism induction by simultaneous transplantation of donor bone marrow with VCA transplantation (Xu et al. 2013). Numerous papers on animal models and clinical trials have confirmed the correlation between chimerism and VCA survival, but some investigators are skeptical (Kashiwagi et al. 1969; Murase et al. 1991). There are many different tolerance induction protocols, including the vascularized whole limb transplantation combined with selected and modified cells line (Ibrahim et al. 2013; Siemionow and Klimczak 2013). It was confirmed that grafting of the vascularized bone containing hematopoietic cells most effectively induces durable chimerism, which correlates with the significant extension of VCA survival (Barth et al. 2011; Bozkurt et al. 2013; Leonard et al. 2013b; Nasir et al. 2010). No scientific reports evaluate the influence of bone marrow transplantation into immunologically privileged regions to induce chimerism and tolerance in the VCA. As pioneers in this research area, we developed an innovative, technical approach for donor BMC transplantation model into the immunoprivileged regions. We also assessed the efficacy of different bone marrow cell transplantation methods on the extension of survival of the allogeneic vascularized skin flaps.

Previous experimental studies confirmed the role of skin components playing the role in the induction of chimerism in the allograft recipients. T lymphocytes, the skin’s natural residents, accompany by dendritic cells and Langerhans’ cells and play the leading role in chimerism induction, which is proportional to the size of the skin component of the VCA (Leonard et al. 2013a). The size of 16 cm^2^ of the skin component of the VCA corresponded to more than half of the skin component included in the entire rat total abdominal wall model of 27.92 cm^2^ (Nasir et al. 2008). The larger skin component involved in VCA transplants induced higher chimerism levels, but the efficacy of induction of the long-lasting tolerance via chimerism is weaker when compared with chimerism induction via vascularized bone marrow transplantation (Arslan et al. 2007; Siemionow and Nasir 2007; Siemionow et al. 2005c). In our study, mixed chimerism in the peripheral blood was composed of T cells (including CD4^+^ and CD8^+^) and B lymphocytes. The highest level of chimerism was observed in all groups on day 7 after transplantation and then decreased over time.

Furthermore, higher chimerism levels in the peripheral blood in the control group negatively correlated with VSA survival, where VSA rejection was triggered two times faster than in Group 4, where the BMC was transplanted into the cerebrospinal fluid via intrathecal administration.

Even more interesting seems to be a higher mixed donor chimerism level in the control group, notwithstanding the donor bone marrow cell transplantation. These results are consistent with earlier results from Siemionow et al. (Nasir et al. 2008). These studies confirmed that the low level of whole chimerism in the peripheral blood induced by the skin allograft could not guarantee prolonged VSA survival. According to the literature and presented results, chimerism less than 10% does not ensure long-term VSA survival (Kanamoto and Maki 2004), in contrast to the survival time of the transplanted organ (Kiyomoto et al. 2006). Our study indicated the importance of different chimeric cell populations playing the role in allograft survival when compared with total chimerism levels below 10%, which is less meaningful. The T cells decline was accompanied by the maintenance of stable values of B lymphocytes. This suggests T cells’ role in the first phase of tolerance induction; however, B cells are responsible for maintaining the second phase of tolerance (Ashour and Seif 2007; Cowan et al. 2012; Parsons et al. 2009). We observed comparable levels of B cells in all groups, and its decrease below 1% was associated with the beginning of the VSA rejection process. This affinity was seen in all groups—the value of B cell fluctuations correlated with a downgrade in T regulatory cells’ level. Many scientific reports confirmed the effect of regulatory T cells (Treg) on the cellular response and the humoral immune response (Eddahri et al. 2006; Fields et al. 2005; Singh et al. 2012). The prolonged deterioration of the B cell’s population in the intrathecal Group reflected prolonged VSA survival (Liu et al. 2009). Previous observations of Prof. M. Siemionow and colleagues’ confirm present assessments (Siemionow et al. 2008; Yazici et al. 2007). The explanation of the lower but stable level of donor peripheral blood B lymphocytes than the values obtained in previous Prof. Siemionow’s study (Siemionow et al. 2005c) was the lack of an adequate pool of progenitor cells in ours study. The vascularized bone marrow is the largest reservoir of these cells. Therefore, the donor B lymphocytes’ level increases significantly after vascularized bone marrow transplantation promoting VSA survival (Klimczak et al. 2006; Lin et al. 2021). This paradox appears due to the delayed redistribution of transplanted cells from immunoprivileged regions, forming a barrier between the external environment and myeloid cells. Moreover, the cells derived from the donor bone marrow migrate and settle in the recipient’s lymphoid tissues, with no peripheral blood presence, reflecting the fundamental values of donor chimerism.

Naturally occurring CD4^+^/CD25^+^ T cells constitute up to 10% of the CD4^+^ T cells in rodents’ peripheral blood. The majority of these cells express transcriptional factor FoxP3 associated with Treg-cell function. It is well known that most of the naturally occurring CD4^+^/CD25^+^ T cells are produced by the thymus as an antigen-primed and functionally mature T cells subpopulation playing the role in the immunosuppression. Also, some of the T cells differentiate from the naive conventional T cells in the periphery and do not require intrathymic preactivation to acquire Treg phenotype and function (Sakaguchi et al. 2008; Yang and Eun 2017). T cells with phenotype CD4/CD25 are essential, particularly in immunoregulation of both the innate and adaptive immune responses, although some reports undermine the validity of these observations (Brazio et al. 2013). In our study, we did not specify CD4^+^/CD25^+^ cells phenotype with FoxP3. The highest level of CD4^+^/CD25^+^ T lymphocytes was observed in the intrathecal Group. The level of CD4^+^CD25^+^ tripled at 35 days after transplantation compared to the levels observed at day 7. The high values of CD4^+^/CD25^+^ cells correlated with a median skin allografts survival and reached the longest survival rate in Group 4 where BMC were injected into the recipient’s cerebrospinal fluid via intrathecal administration. The values of CD4^+^/CD25^+^ cells observed in the other groups were significantly lower, represented by shortened vascularized flap survival. Besides, the kinetic changes of CD4^+^/CD25^+^ lymphocytes during the follow-up period were similar in all groups except Group 4. In the intrathecal Group, we first observed an increasing trend of up to 35 days and decreased thereafter. Cerebrospinal fluid offers the most favorable conditions, guarantees survival and migration of progenitor cells compared to other immunologically privileged regions (Pilat et al. 2010; Zhu et al. 2002). Recent in vitro studies documented that T lymphocytes can enter the central nervous system directly by crossing the endothelial blood–brain-barrier or enter into the cerebrospinal fluid via the choroid plexus (Nishihara et al. 2020; Strazielle et al. 2016). Thus, intrathecal delivery of BMC can results in “inverse” trafficking of transplanted cells into the systemic circulation, thus enhancing peripheral blood chimerism. This notion can be supported by studies revealing the presence of functional meningeal lymphatic system located in the dura mater and enables cerebrospinal fluid, and the immune cells to drain back to the deep cervical lymph nodes (Louveau et al. 2015). Therefore, allogeneic antigens present on the transplanted BMCs can trigger naïve T lymphocytes residing in the lymph nodes for differentiation into the functional CD4^+^CD25^+^ Tregs that are engaged in the suppressing of the immune responses towards transplanted allografts (Paiva et al. 2013; Sakaguchi et al. 2008).

Treg cells are critical for maintaining tissue (immune) homeostasis mainly by exerting suppressive function on the naïve or effector T cells. In transplantation tolerance, Tregs can function to regulate alloimmune response through several mechanisms, including secretion of the immunosuppressive cytokines (TGF-β and IL-10), modulation of the antigen-presenting cell (APC) function (e.g., downregulation of expression of dendritic cells co-stimulatory molecules, or by direct suppression of CD8^+^ and CD4^+^ effector cells by cell-to-cell contact (Rahhal et al. 2009; Sakaguchi et al. 2008). Experimental studies on spinal Treg cells delivery and intrathecal Treg-dependent IL-35 delivery proved that these cells reduced pain associated with encephalomyelitis in the experimental autoimmune encephalomyelitis mice model. This was associated with the upregulation of IL-10 expression (Duffy et al. 2019). Functional activity of Treg assessed in osteomyocutaneous VCA mouse model showed that depletion of Treg at day 30 after allograft transplantation is leading to allograft rejection. Moreover, Treg from tolerant mice showed more significant suppressive potential and the ability to rescue allografts from rejection, thus confirming that circulating Treg’s are crucial for tolerance induction in VCA in the early post-transplant period (Anggelia et al. 2021). This study also confirmed that an elevated level of IL-10 was associated with the protolerogenic function of Treg.

Moreover, two more mechanisms’ are responsible for increased cell survival in the immunoprivileged compartments: MHC APO-1/FasL expression and low level of APCs due to the low lymph drainage. Moreover, CD4^+^/CD25^+^ cells decreased at the observation time in both intragonadal and intrathecal groups, confirming these compartemnets as the preferable injection sites leading to the prolonged VSA survival (*p* = 0.002 and *p* = 0.001) when compared with the control group. Moreover, in the intrathecal Group, the increased number of the Treg lymphocytes when compared with a control group has established the intrathecal compartment with cerebrospinal fluid as the preferred site for BMC transplantation which was confirmed by triggering of the CD4/CD25 cells in the peripheral blood. We observed the statistically significant difference in the decreased CD4/CD25 cells’ value in the control and intracapsular Groups (*p* < 0.02, *p* < 0.03). Furthermore, immunoprivileged compartments guaranteed the transplanted BMC protection against annihilation. Furthermore, the role of B cell chimerism should be mentioned, since considering the functional similarities of regulatory B cells and regulatory T cells allows to assume that increased B-cell chimerism in the intrathecal BMC group enhanced the immunosuppressive effect of Tregs and extended VCA survival (Lee et al. 2014).

The engraftment process was supported by selective blockage of alloreactive T cells by anti-alfa/beta-TCR antibodies preserving tolerogenic potential of gamma/delta T lymphocytes (Shevach 2006). The first clinical trials with intrathecal BMC transplantation reported promising outcomes (Gahrton et al. 1991), however, more clinical trials and investigations are needed to support routine application of this protocol.

The transplantation procedure was not burdened by the GVHD, regardless of the privileged site of BMC transplantation. This observation is consistent with previous reports, which assessed the combined application of the immunosuppressive protocol with BMC transplantation in the CTA model (Siemionow et al. 2012; Xu et al. 2013). The lack of GVHD can be explained either by the selective immunosuppressive potential of the alpha/beta TCR protocol or by the use of CsA. The CsA treatment inhibits predominantly production of IL-2, resulting in a lack of T cell proliferation and thereby facilitates donor BMC engraftment. A 7-day immunosuppression protocol combined with BMCs transplantation creates an immune-privileged window for the Treg cells migrations to the immunologically reactive regions (Janssens et al. 2003; Walch and Lakkis 2014). Engraftment of donor cells into both the lymphoid and non-lymphoid organs of the recipient presented the Starzl innovatory chimerism theory (Starzl 1971) indicating that only constant contact of the donor cells with the recipient lymphoid organs warrants transplantation tolerance induced by chimerism. The immune protection mechanism is based on the isolation of foreign antigens by the mechanical and immunological barrier created by the recipient’s immunoprivileged sites. This development is critical because the robust migration of the APC from the VCA donor in the initial phase of transplantation triggers a significant immune response in the recipient (Hoffman et al. 1989). The immunoprivileged regions possess several innate mechanisms such as an anatomical barrier, increased Treg cell activity, increased activation of IL-10 and TGF-α, and membrane FasL factor (D’Alessio et al. 2001). These mechanisms steered activated Treg cells to apoptotic death. Due to these reasons, allograft acceptance without the need for an immunosuppressive “umbrella” may have occurred (Krajewska et al. 2006).

The limitation of this study is the lack of functional assessment on the correlation of the levels of CD4^+^/CD25^+^ cells with increased graft survival in the intrathecal Group. However, this study was focused on the assessment of the best-immunoprivileged compartment for BMC delivery which would correlate with VCA survival. Thus, the current study is considered as a pilot study searching for the best-immunoprivileged tissue/organ which will be chosen for the further studies assessing the impact of CD4^+^/CD25^+^ cells on functional immune activity and donor-specific tolerance in the allograft recipients. However, despite these limitations, the present study adds a novel observation of application and delivery of the BMC supportive therapy into immunoprivileged compartments as a potential tool for tolerance-inducing strategies, an extension of VCA survival.

## Conclusions

Our protocol is short, selective, and minimally toxic and creates optimal conditions for implantation of both cells of foreign origin and allows for the coexistence of these cells in one organism (Nikolic et al. 2010; Siemionow and Klimczak 2009). The assurance of the dynamic correlation between the donor and recipient cells illustrates the chimerism-inducing phenomenon, limiting the need for lifelong immunosuppressive therapy (Leonard et al. 2012). Seven days of immunomodulatory therapy with the donor bone marrow cells sensitization in the immunologically privileged regions created the favorable conditions for allogeneic BMT and subsequent intra-bone marrow engraftment (Siemionow and Klimczak 2010). The presence of donor chimerism in the recipient’s peripheral blood and lymphoid organs promotes survival of the vascularized skin allografts. Supportive intrathecal (to the cerebrospinal fluid) transplantation of the BMC is more effective in extending the survival of the vascularized skin allografts compared to the BMC injection into the immuno-privileged regions under the kidney capsule or into the testis. Intrathecal transplantation is associated with the higher levels of the subpopulation of CD4^+^/CD25^+^ T cells in the peripheral blood which is corresponding with a stable levels of the donor B cells RT1^a^/CD45RA population. These results are promising and may be applied as a novel immunomodulatory therapy for tolerance induction in CTA (Oh et al. 2020).

## Data Availability

All data generated in this study are included in the manuscript. Moreover, they are available for presentation upon request.
